# SANSparallel: interactive homology search against Uniprot

**DOI:** 10.1093/nar/gkv317

**Published:** 2015-04-08

**Authors:** Panu Somervuo, Liisa Holm

**Affiliations:** 1 Institute of Biotechnology, University of Helsinki, PO Box 65, Finland; 2 Department of Biosciences, University of Helsinki, PO Box 65, Finland

## Abstract

Proteins evolve by mutations and natural selection. The network of sequence similarities is a rich source for mining homologous relationships that inform on protein structure and function. There are many servers available to browse the network of homology relationships but one has to wait up to a minute for results. The SANSparallel webserver provides protein sequence database searches with immediate response and professional alignment visualization by third-party software. The output is a list, pairwise alignment or stacked alignment of sequence-similar proteins from Uniprot, UniRef90/50, Swissprot or Protein Data Bank. The stacked alignments are viewed in Jalview or as sequence logos. The database search uses the suffix array neighborhood search (SANS) method, which has been re-implemented as a client-server, improved and parallelized. The method is extremely fast and as sensitive as BLAST above 50% sequence identity. Benchmarks show that the method is highly competitive compared to previously published fast database search programs: UBLAST, DIAMOND, LAST, LAMBDA, RAPSEARCH2 and BLAT. The web server can be accessed interactively or programmatically at http://ekhidna2.biocenter.helsinki.fi/cgi-bin/sans/sans.cgi. It can be used to make protein functional annotation pipelines more efficient, and it is useful in interactive exploration of the detailed evidence supporting the annotation of particular proteins of interest.

## INTRODUCTION

Recent years have witnessed a remarkable growth in the number of sequences. This has made database searches (1–4) take longer and longer and forced free computing services and pre-computed databases to close down or resort to crowd-sourcing (5–7). SANSparallel is a web server that takes protein sequences as input and returns an approximate set of closest sequence neighbors in the blink of an eye. At the core of our web server is a fast database search engine that only takes a fraction of a second to compare a query protein against 90 million sequences in Uniprot (8). SANSparallel is a re-implemented, improved and parallelized version of our previous suffix array neighborhood search (SANS) algorithm (9). It belongs to a new generation of fast database search programs indexing the database so that short words (seeds) matching to the query can be found efficiently and independent of database size (10–15). Similar sequences can then be identified by seed extension or by counting how many seeds match one database protein. Suffix arrays bring the advantage that seed length can be adapted to increase selectivity. On the other hand, spaced seeds and reduced alphabets have been introduced to increase sensitivity (16). Programs implementing these techniques are orders of magnitude faster than BLAST. However, it is hard to match BLAST's sensitivity. These approaches are very suitable for mapping problems, where the match is very close and gives a clear signal. We have found previously that the approach works reliably in protein database searches above 50% sequence identity (9). Here, we present more benchmarking and show that SANSparallel is highly competitive in comparison with recently published programs.

## MATERIALS AND METHODS

### System architecture

SANSparallel runs as a client and a server. The server holds the database in memory and performs the search. We have a separate server for each database. Client processes connect to the server and transmit the query sequence to the server and the result to the user. Multiple clients can connect to the server. Concurrent clients are served one query at a time in round-robin fashion. From the users’ perspective this means that the time it takes to process a query increases linearly with server load, but all users experience similar speed. Linearity of response times was maintained up to at least 100 concurrent clients (data not shown).

**Figure 1.**
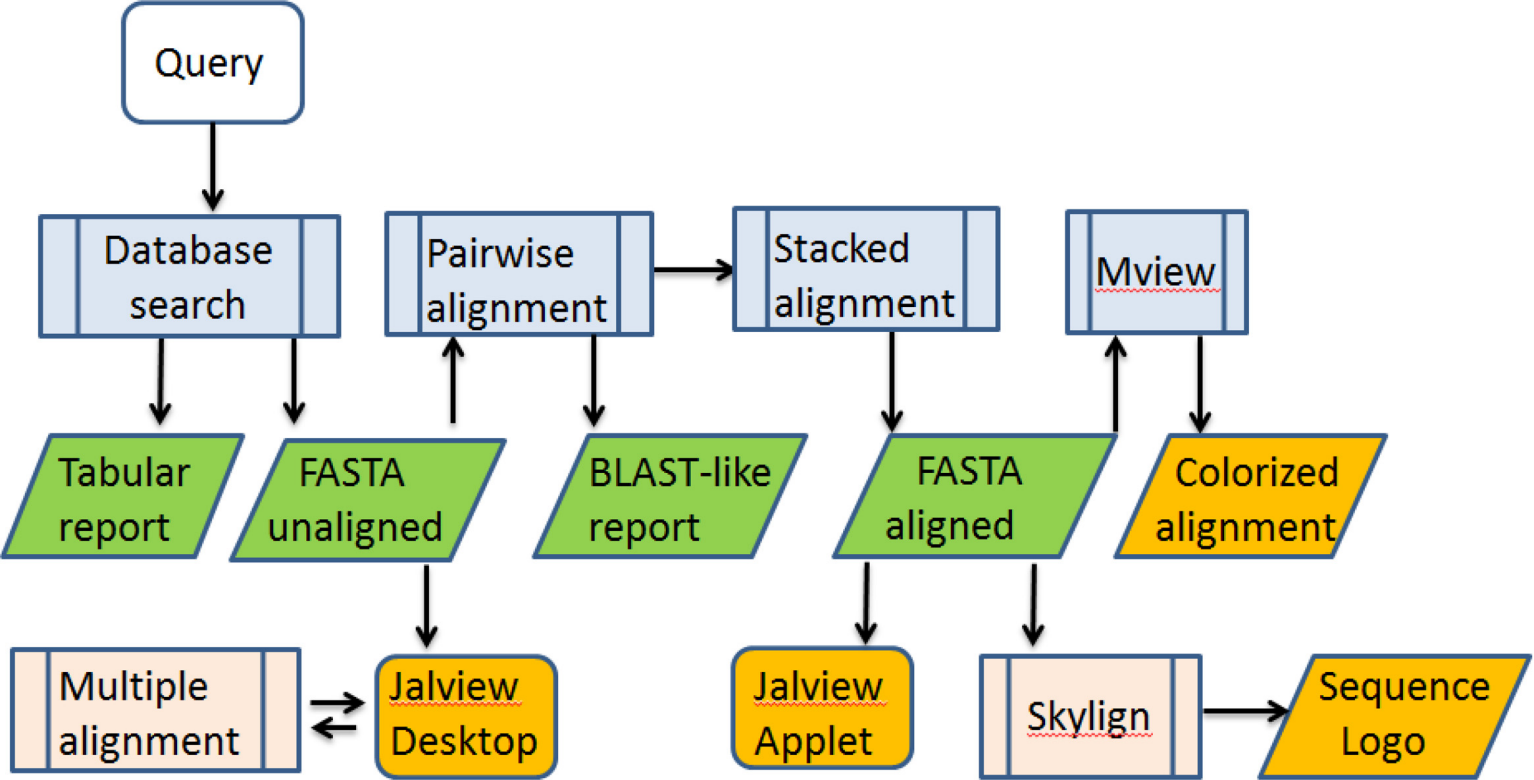
Flowchart of the SANSparallel web server. Computations done by the web server are blue. Results sent to the user include textual outputs (green) and alignment visualizations (orange). Multiple alignment (instantiated from Jalview Desktop) and sequence logo computations utilize third party resources in the cloud (pink).

Underlying the web server is a CGI script which calls the client program with appropriate options and post-processes the database search results into the desired output format (Figure 1). Some processing steps use third-party software. The primary result from SANSparallel is a set of sequence-similar proteins retrieved from the database. Pairwise alignments between this set of sequences and the query sequence are generated using FASTA (17). The same program is used to output a BLAST-like report. The pairwise alignments are stacked against the query sequence, omitting insertions to generate gapped alignments. The stacked alignment can be colorized by Mview (18) or sent to Skylign (19) to generate a sequence logo. Aligned or unaligned sequences can be output in FASTA format and sent to Jalview (20) for alignment visualization and editing. Our server does not provide multiple sequence alignments as this can be very time consuming. Instead, multiple sequence alignments can be requested from Jalview Desktop's web service menu. The response of the server is immediate and no user data or results are stored on disk except for results viewed with the Jalview applet, which requires file input.

SANSparallel was developed in a Linux operating system and parallelized using openmpi. The web server runs on a cluster of computers with 500-Gb memory and 64 cores. SANSparallel was written in Fortran using legacy code from SANS (9), socket communications in C and the CGI script in Perl. Storage of the database in memory and additional work space take about 9 bytes per amino acid.

### Database search algorithm

SANSparallel is a re-implemented, improved and parallelized version of the suffix array neighborhood search algorithm SANS (9). Briefly, the algorithm accumulates a vote for database proteins that are found within a window of the position where a suffix of the query sequence would be inserted in the suffix array of the database. Database proteins with the highest votes are collected and, optionally, aligned and resorted by the alignment score. The following changes were introduced: (i) a binary search to find the suffix array insertion position replaces the original mergesort. This enables searching single query sequences instead of the original batch processing. (ii) Votes are summed over diagonal bands rather than the whole protein. This improves selectivity. A similar strategy is used in the FASTA algorithm (17). (iii) Alignments are computed by dynamic programming in a diagonal band. This replaces the original program's greedy algorithm to combine high-scoring segment pairs. e-values are computed from the alignment score using Karlin–Altschul statistics (21). (iv) There is a positive but not perfect correlation between the vote and pairwise alignment score. An option was added to moving down the sorted list of database proteins until the H^th^-best alignment score remains stable. This results in more closely similar hits in the output. (v) The program was parallelized using MPI (Message Passing Interface). We chose a micro parallelization strategy in order to achieve fast response times for a single query. One node is reserved for communication with the client. The other nodes are dedicated to the database search. Each node works on a section of the database. The database search nodes go into hibernation when traffic is low. Search speed increased linearly up to 8–16 nodes; above 32 nodes there was not enough work to match communication overheads (data not shown).

### Databases

The Uniprot, UniRef90, UniRef50 and Swissprot databases are downloaded monthly from ftp.ebi.ac.uk. The sequences of Protein Data Bank entries are taken weekly from the Dali server (22).

### Benchmark data sets

The server was benchmarked using the same test set and database as in (9). The test set consists of 4174 predicted proteins of *Dickeya solani*, an emerging plant pathogen (23). The reference database is Uniprot frozen in 2012, which did not yet contain *D. solani*. The reference set of TRUE hits was generated using SSEARCH (17) and an e-value cutoff of 1.0. Others have observed before us that implementations differ between programs and e-values are not directly comparable between programs (12). Therefore programs being evaluated were asked to output 1000 best hits. Hits found in the reference set were counted as true positives. Most programs compute an e-value for the hits, which operationally eliminates false positives. The hits were also subdivided into bins according to the sequence identity of the pair in the reference set. The wall-clock time to process the test set was also recorded to compare speeds.

BLAST, UBLAST, LAMBDA, RAPSEARCH2 and SANSparallel are natively parallel. LAST was run with GNUparallel using blocksize 36 000. We used pre-compiled LAMBDA v0.4.7 which could not output more than 500 hits per query; this bug was fixed but a new version was not available in time for our benchmarks (Hannes Hausdewell, personal communication). All software used were 64-bit versions except UBLAST of which only a 32-bit version is freely available. Due to 32 bits, reference database needed to be split into several chunks in order to index it. Also BLAT required the reference data to be split into several segments in order to work. The e-value threshold was set to 1.0 in all software where this option was available. In LAST, the score threshold was calculated to correspond to e-value 1.0 and was set accordingly. LAST parameter –m 500 was used in order to get more hits. Otherwise default parameters were used.

## RESULTS

### Benchmarking

**Figure 2.**
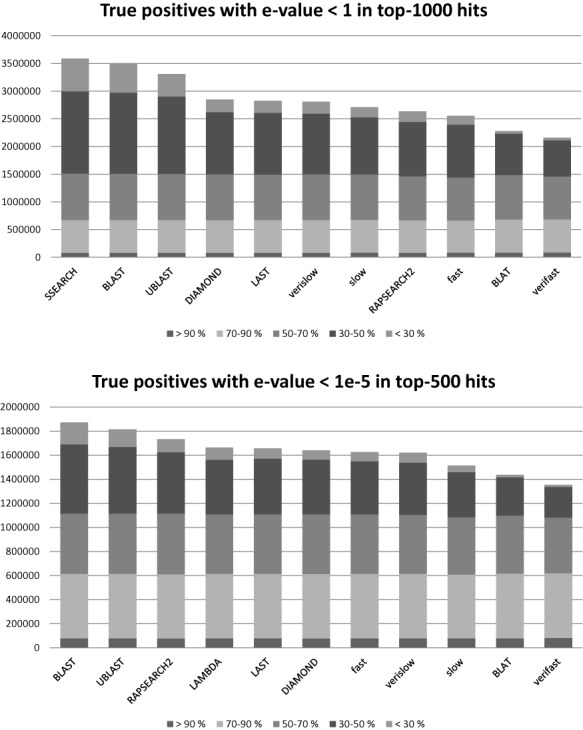
Benchmark results showing the number of true positives detected in the top-1000 hits and top-500 hits binned by sequence identity.

We tested SANSparallel against BLAST (1), UBLAST (14), LAMBDA (12), LAST (13), DIAMOND (15), BLAT (10) and RAPSEARCH2 (11) using the same benchmark as in (9). Four modes of SANSparallel (verifast, fast, slow and verislow) were used which differ in the depth and speed of the search. LAMBDA outputs maximally 500 hits, therefore comparisons are shown for 1000 hits and 500 hits. The performance of all methods is quite similar above 50% sequence identity, differences are mainly seen in the detection of remote homologs below 50% sequence identity (Figure 2). The sensitivity of UBLAST is closest to BLAST. RAPSEARCH2 and BLAT are both slower and less sensitive than at least one competing method. Some aligners have tunable parameters whereby one can arbitrarily trade speed for sensitivity. Also SANSparallel gets faster when fewer hits are output (Table 1). Considering both speed and sensitivity, a group of four methods emerges with small differences between them: SANSparallel fast mode, DIAMOND, LAMBDA and LAST. Fast is the default mode in the SANSparallel web server.

**Table 1.** Speed comparison of database search programs: time taken to search 4174 queries of the *Dickeya solani* benchmark

Program	Hits	Cores	Time (s)	Relative speed
verifast	100	16	62	5903
fast	100	16	65	5631
verifast	500	16	111	3298
verifast	1000	16	170	2153
fast	500	16	178	2056
LAMBDA	500	16	216	1695
slow	100	16	235	1558
fast	1000	16	324	1130
LAST	1000	16 ^a^	327	1119
slow	500	16	406	902
DIAMOND	1000	16	446	821
slow	1000	16	612	598
verislow	500	16	624	587
verislow	1000	16	792	462
verifast	1000	1	1009	363
UBLAST ^b^	1000	16 ^a^	1310	279
RAPSEARCH2	1000	16	1469	249
LAMBDA	500	1	2052	178
LAST	1000	1	2957	124
fast	1000	1	3297	111
SANS^c^	1000	1	3809	96
BLAT ^b^	1000	1	4307	85
slow	1000	1	5015	73
verislow	1000	1	7094	52
RAPSEARCH2	1000	1	18761	20
UBLAST ^b^	1000	1	28399	13
BLAST	1000	16^a^	32149	11
BLAST	1000	1	366046	1

^a^GNUparallel.

^b^Database split to chunks (UBLAST: 19, BLAT: 5) due to program's size limit.

^c^Serial implementation (9).

### User interface

#### Inputs and outputs

**Figure 3.**
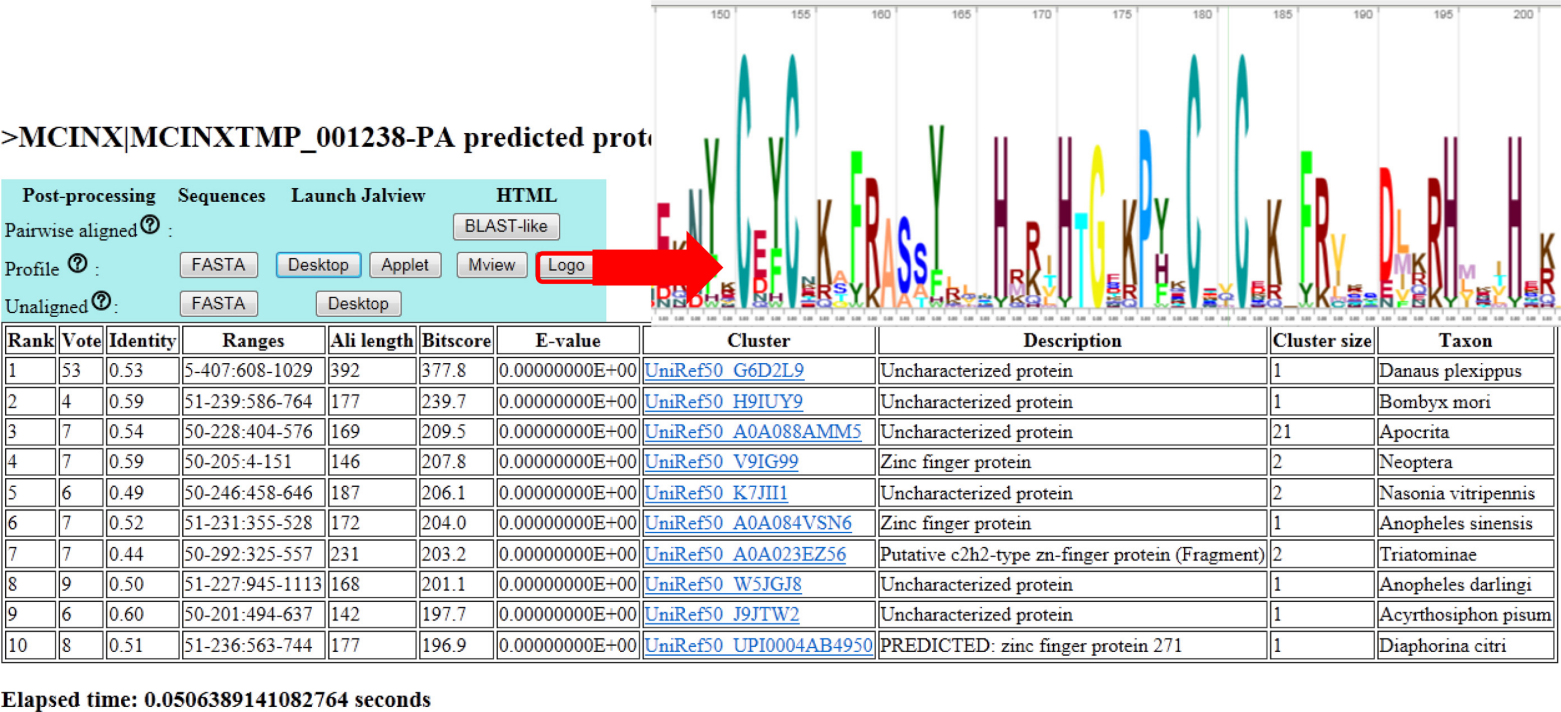
Example output.

The website is free and open to all and there is no login requirement. The input to the server are FASTA-formatted sequences. One or multiple query sequences can be submitted in one request. The user can also choose the maximum number of hits to be output (H), the database to be searched (Uniprot, UniRef90, UniRef50, Swissprot or PDB) and a search protocol. The protocols are pre-set parameter combinations: (i) verifast mode reports H proteins with the highest vote; no alignments are computed. (ii) Fast mode is like the previous mode but reports alignment scores. (iii) Slow mode inspects 2H proteins with the highest vote and sorts them by alignment score. (iv) Verislow mode maximizes accuracy when H is small. It always inspects 4000 proteins with the highest vote and sorts them by alignment score. The vote threshold of verifast mode is set so that the false positive rate is 1–2% in our benchmark. The other modes only report hits with an e-value below 1. Figure 3 illustrates the search result for a predicted protein from the butterfly *Melitaea cinxia* (24), which the cgi-script generated in 51 milliseconds. The primary output of the server is a tabular report of the hits with links to different output options (Figure 3). For example, we generate stacked alignments that are automatically loaded to Jalview (20) for alignment editing/visualization or to Skylign (19) for drawing sequence logos. Jalview Desktop is a standalone Java application that can be downloaded from http://www.jalview.org/download. The Jalview applet is launched from our website which must be added to the user's list of trusted sites as instructed in the tutorial (http://ekhidna2.biocenter.helsinki.fi/sans/Tutorial.html#exercises). Skylign outputs HTML5 which works on modern web browsers.

#### Programmatic access

SANSparallel can be used for both interactive and high-throughput analyses. All input and output options of the cgi-script can be included in the URL as explained in the web tutorial (http://ekhidna2.biocenter.helsinki.fi/sans/Tutorial.html#external). Thus, another web server can link to SANSparallel to retrieve information about the sequence neighbors of a particular protein. Another use of SANSparallel is in high-throughput functional annotation of proteomes or transcriptomes. For example, the web tutorial demonstrates (http://ekhidna2.biocenter.helsinki.fi/sans/Tutorial.html#perl) how to build a simple annotation pipeline where (i) the predicted protein sequences (in FASTA format) are sent to the server, (ii) the result is parsed and filtered, (iii) the best informative hit is selected as a source of annotation of the query sequence and (iv) a summary table is generated which reports the predicted annotation of each query protein and links its sequence back to SANSparallel so that anyone interested can study the evidence for the prediction interactively. Finally, it is possible to download the client-server programs in source code (http://ekhidna2.biocenter.helsinki.fi/sans/download/) and run the programs locally on local databases.

## DISCUSSION

We have improved and parallelized the suffix array neighborhood search algorithm SANS (9). Our benchmarking results were in line with previously published comparisons identifying UBLAST as sensitive and LAST and LAMBDA as fast. SANSparallel is competitive with DIAMOND, LAST and LAMBDA. All these programs are based on similar principles but with different implementations. Benchmarking showed that they miss few hits when sequence identity is above 50% but fall behind BLAST when sequence identity gets lower (Figure 2). Future work will focus on improving sensitivity by increasing the sequence space coverage of the seeds. The speed of SANSparallel depends on the amount of output (Table 1). LAST has no direct control on the number of hits, but this is influenced by the –m parameter for the uniqueness of seeds in the database (13). DIAMOND (15) and LAMBDA (12) are designed for batch processing of large query sets like the original SANS algorithm (9). The SANSparallel server supports both interactive analysis of individual queries and high-throughput analysis of genomes or transcriptomes. It is simple to link to other tools, as inputs and outputs are FASTA-formatted sequences or alignments. Much can be learned by studying groups of homologous proteins instead of individual proteins. Evolutionary conservation sharpens the signal for function (25,26), secondary structure (27) and deeper homology detection (1). SANSparallel facilitates such analyses by retrieving homologs from the database and performing an alignment. It is so fast that the user can change output formats, search parameters or the database interactively. Speed opens up new ways to operate. For example, functional annotations of genomes could be updated on demand, database clustering need not store all-against-all search results on disk, and sequence similarity based data integration could be done on the fly.
